# Synthesis of Silver and Gold Nanoparticles from *Rumex roseus* Plant Extract and Their Application in Electrochemical Sensors

**DOI:** 10.3390/nano11030739

**Published:** 2021-03-15

**Authors:** Meryam Chelly, Sabrine Chelly, Rayhane Zribi, Hanen Bouaziz-Ketata, Radhouane Gdoura, Nehru Lavanya, Ganesan Veerapandi, Chinnathambi Sekar, Giovanni Neri

**Affiliations:** 1 Laboratory of Toxicology-Microbiology Environmental and Health, LR17ES06, Sfax Faculty of Sciences, University of Sfax, BP 1171, Sfax 3000, Tunisia; chelly.meryam@gmail.com (M.C.); sabrine.chelly@yahoo.com (S.C.); hanenktata@yahoo.fr (H.B.-K.); gdoura.radhouane@gmail.com (R.G.); 2 Department of Engineering, University of Messina, C.da Di Dio, I-98166 Messina, Italy; zribi.rayhane@gmail.com; 3 Department of Bioelectronics and Biosensors, Alagappa University, Karaikudi 630004, India; lavan153@gmail.com (N.L.); g.veerapandiavc@gmail.com (G.V.); sekar2025@gmail.com (C.S.)

**Keywords:** silver and gold nanoparticles, *Rumex roseus*, plant extract, electrochemical sensors, hydrogen peroxide, riboflavin

## Abstract

The room-temperature synthesis of silver (AgNPs) and gold (AuNPs) nanoparticles from aqueous solution of AgNO_3_ and HAuCl_4_ respectively, using *Rumex roseus* (RR) plant extract as a reducing agent, is reported here for the first time. The nanoparticles obtained were characterized by UV-Vis spectroscopy, transmission electron microscopy (TEM) and dynamic light scattering (DLS). The formation of nanoparticles with spherical-shaped morphology was verified by TEM and confirmed by UV-Vis spectroscopy through the analysis of Ag and Au plasmon resonance peak and DLS measurements. New electrochemical sensors have been developed by employing the synthesized Ag and Au nanoparticles as modifiers of glassy carbon electrode (GCE) and screen-printed carbon electrode (SPCE), respectively. The AgNPs-modified GCE was investigated for the electrochemical determination of hydrogen peroxide (H_2_O_2_). Further enhancement of electrochemical performances was obtained using a nanocomposite made of AgNPs and reduced graphene oxide (rGO)-modified GCE. The AuNPs-SPCE sensor was instead tested in the electrochemical sensing of riboflavin (RF). To our knowledge, this is the first paper reporting *Rumex roseus* plant extract as a source for the synthesis of metal nanoparticles and their use for developing simple, sensitive and reliable electrochemical sensors for H_2_O_2_ and RF.

## 1. Introduction

It is well known that metals at the nanometer scale may offer many advantages in the electrochemical field, due to their high surface energy and conductivity which help in catalyzing electrochemical reactions and enhance charge transfer at the electrode surface. Thus, within the primary focus on the development of electrochemical sensors for electroactive substances, a number of different metal nanoparticles (NPs) have been proposed and applied as electrode modifiers [1,2]. In this respect, a special attention has been given to silver and gold nanoparticles (named AgNPs and AuNPs, respectively) due to their large effective surface area, excellent conducting capability, fast mass and charge transport and effective electrocatalytic characteristics [3,4]. Therefore, the preparation of these metal nanoparticles by means of simple and reliable synthesis processes is of great importance nowadays [5,6,7].

Among the large variety of methods proposed for the preparation of Ag and Au nanoparticles, a great deal of attempts have been put into their synthesis by using plant extracts [8,9,10,11,12,13]. This derived from the presence in the extract of a number of different antioxidant molecules such as polyphenols, which are able to reduce metal ions into metal species. It is noteworthy that hydroxyl and/or ketonic groups of these compounds are also able to bind the as-formed metal nanoparticles, too, providing an enhanced stability against their tendency to agglomeration. A lot of plants have been reported in the literature, providing the basis for processes devoted to the synthesis of Ag and Au nanoparticles. For example, Naznin et al. exploited the properties of *Black Tea* leaf extract, known to be rich in antioxidant molecules, for synthesizing Ag and Au nanoparticles [14]. Rodríguez-León et al. prepared silver nanoparticles from silver nitrate using extracts of *Rumex hymenosepalus* [15].

In the present study, the synthesis and characterization of Ag and Au NPs using plant extract of *Rumex roseus* (*R. roseus*, RR) has been described. *Rumex roseus* is a wild local Tunisian plant. In a previous work, we have evaluated its composition, identifying a number of phytochemicals [16]. Among the others, this plant contains a large amount of phenolic compounds such as luteolin and ferulic acid. These molecules are potentially strong reducing agents due to the presence of numerous OH^−^ and C=O groups. However, so far, to the best of our knowledge, this is the first report in the literature on the synthesis of metal nanoparticles using the extract of *Rumex roseus*.

Here, we have investigated the sensing properties of the synthesized AgNPs and AuNPs with the aim to develop electrochemical sensors by using these nanoparticles as modifiers of glassy carbon electrode (GCE) and screen-printed carbon electrode (SPCE), for the monitoring of hydrogen peroxide (H_2_O_2_) and Riboflavin (Vitamin B2, RF), respectively. H_2_O_2_ is an important molecule within the human body, playing various roles in physiological processes, where it regulates cell growth, whereas, at high levels, H_2_O_2_ can be detrimental, causing inflammation and cell damage [17]. Riboflavin is essential for the development of infant tissues, the function of the muscles, heart, the nervous system and mental activity [18]. This vitamin is also involved in carbohydrate, lipid and protein metabolism, red blood cell formation, respiration, antibody production and normal infant growth [19]. H_2_O_2_ cannot be determined at bare glassy carbon electrode, and the presence of a modifier explicating good catalytic properties (e.g., PdNPs) is necessary for obtaining an acceptable electrochemical response [20]. Kadara et al. developed an unmodified SPCE for the determination of riboflavin, applied to the analysis of food products [21]. To introduce further improvements, the modification of electrodes is carried out by utilizing various nanomaterials, including metal oxides and noble metals, primarily to overcome the intrinsic limitations of bare electrodes, e.g., low sensitivity, scarce selectivity and poor stability [1,2,3,22].

We have been also involved for a long time in the modification of material electrodes for improving their performances towards hydrogen peroxide and several vitamins, including riboflavin [23,24,25,26]. For example, Lavanya et al. fabricated a hydrogen peroxide biosensor based on Ni-doped SnO_2_ nanoparticles for H_2_O_2_ sensing [23]. For promoting the sensitivity towards the same analyte, Leonardi et al. used Pt–TiO_2_/reduced graphene oxide nanocomposites [24]. Riboflavin and other vitamins’ monitoring also benefit from the enhanced performances of modified electrodes with Cu- and Cr-doped SnO_2_ nanoparticles [25,26].

In the course of these studies, it has been found that the electrochemical characteristics of metals or metal oxides are remarkably improved by depositing them on reduced graphene oxide (rGO) [24]. These observations are in agreement with many literature reports; rGO, consisting of single planar sheets densely packed in a honeycomb crystal lattice, is an ideal substrate for tethering metal nanoparticles and making more efficient electrochemical sensors [27,28,29,30]. Then, in order to enhance the electrode performances, AgNPs synthesized here were also dispersed on rGO to form AgNPs-rGO composite and followed by evaluation of its efficacy in the modification of a GCE for H_2_O_2_ electrochemical sensing.

Despite the advances in the field of electrochemical detection, there is still much study to do regarding new materials and techniques in sensor design and manufacturing to be proposed for making further improvements. Then, through the optimal combination of metal nanoparticles and electrochemical platforms, we have tried to obtain substantial improvement in the electroanalytical determination of the analytes object of this study.

## 2. Materials and Methods

### 2.1. Chemical Reagents

The reagents and solvents used in this paper are as follows: distilled water (Millipore Water Purifier, Merck, Darmstadt, Germany), methanol (99.8%, Sigma-Aldrich, Darmstadt, Germany), AgNO_3_ (≥99.0%, Sigma-Aldrich), HAuCl_4_ (≥99.9%, Sigma-Aldrich), isopropanol (99.5%, Merck), ethanol (99.8%, Sigma-Aldrich), H_2_O_2_ (30% (*w*/*w*) in H_2_O, Sigma-Aldrich), riboflavin (≥98%, Sigma-Aldrich), phosphate buffer solution (PBS, Thermo Fisher Scientific, Waltham, MA, USA) and ferrocyanide (K_4_[Fe(CN)_6_], 99%, Sigma-Aldrich). The above-mentioned reagents have been used without further purification.

### 2.2. Synthesis of Ag and Au NPs

The preparation of *Rumex roseus* extract, NPs and rGO is described below. The extract of *Rumex roseus* was obtained as follows: first, *Rumex roseus* whole plant was harvested in February 2019, cleaned with tap water and dried under shade for one week. The dried plant was ground into coarse powder by a mechanical grinder. The coarse powder was then subject to maceration with methanol (100%) for 24 h (for 3 times) and filtered. The filtrates were pooled and the solvent was removed using a rotary evaporator. The concentrated extract was stored at 4 °C. More details about this procedure can be found in Reference [16].

**Scheme 1:**
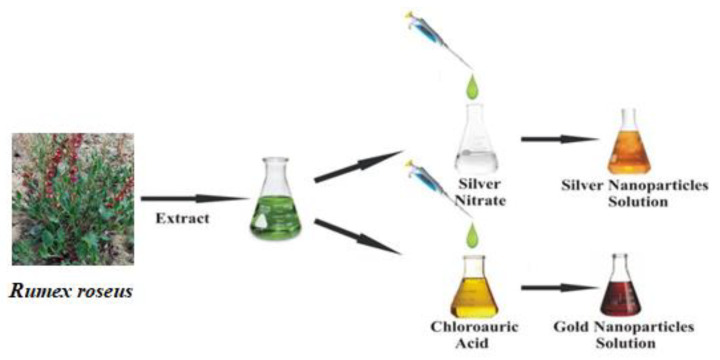
General procedure for the preparation of silver (Ag) and gold (Au) nanoparticles (NPs).

AgNPs and AuNPs were obtained by adding, drop by drop, 5 mL of methanolic *Rumex roseus* extract (6.67 mg/mL) to 45 mL of 1 mM aqueous solution of AgNO_3_ or HAuCl_4_ respectively, at room temperature, under stirring conditions for 2 h (Scheme 1). The reduction of metallic ions to Ag and Au nanoparticles was confirmed by visual color change of the original solutions of AgNO_3_ or HAuCl_4_ respectively, and by UV-Vis measurements with reaction time.

rGO was synthesized by the method as described in our earlier report [30]. The formation of rGO from GO was confirmed by Fourier Transform Infra-Red spectroscopy (FTIR) and Raman measurements. The preparation of AgNPs-rGO composite was carried out as follows: 1 mg of rGO was dispersed in water by sonication for 30 min and then 1 mg AgNPs were added into the solution under stirring. Afterwards, the resulting suspension was kept in a microwave oven at 600 W for 10 min, and then cooled to room temperature. The sample was centrifuged at 3000 rpm for 30 min and dried at room temperature.

### 2.3. Characterization

UV-Vis measurements were performed by using a Jasco V-570 Lambda 1050 (PerkinElmer, Boston, MA, USA) instrument. Transmission electron microscopy (TEM) analysis was carried out with a Philips CM12 (Philips, Eindhoven, The Netherlands) instrument, depositing a drop of the synthesized samples dispersed in isopropanol on the copper grid coated with a carbon layer. The particle size distribution was measured using a dynamic light scattering (DLS) Zetasizer Nano ZS ZEN3600 (Malvern, Worcestershire, UK) instrument.

### 2.4. Modified Electrodes Fabrication

Modified glassy carbon electrodes (GCE) were prepared by using a bare GCE (~3 mm in diameter, supplied by CH Instruments, Shanghai, China), polished with alumina powders (1.0, 0.5 and 0.3 µm) and then rinsed with ethanol and distilled water and dried at room temperature (RT). Then, 10 μL of the suspension of nanoparticles (AgNPs and AgNPs-rGO) were drop-coated on the electrode, for preparing the AgNPs- and AgNPs-rGO-modified electrodes respectively, and left to dry at RT. Electrochemical measurements were performed using a conventional three-electrode cell with a platinum plate as a counter electrode and an Ag/AgCl as the reference electrode. Electrochemical measurements were carried out by using an electrochemical station CHI 660e Electrochemical Analyzer (CH instruments, Shanghai, China).

Modified screen-printed carbon electrodes (SPCE) were prepared by using screen-printed carbon electrode DRP-100 (DropSens, Asturias, Spain) as an electrochemical platform. The bare SPCE is constituted of a 4 mm diameter carbon working electrode, a silver pseudo-reference electrode and a carbon auxiliary electrode. To modify the bare SPCE, a solution containing the nanoparticles was directly drop-casted onto the surface of working electrodes and left at room temperature to dry until further use. Electrochemical measurements with SPCE sensors were carried out by using a DropSens µStat 400 Potentiostat (DropSens, Spain) empowered by Dropview 8400 software for data acquisition.

### 2.5. Electrochemical Tests

Cyclic voltammetry (CV) tests were carried out in PBS at scan rate of 50 mV/s, in the presence of the investigated analytes (H_2_O_2_ = 0–2500 µM; RF = 0–500 µM). Calibration curves were obtained by plotting the peak current vs. analyte concentration in the potential range −0.8 to +0.6 V for H_2_O_2_, and −0.8 to 0.0 V for RF. The sensitivity was computed as the slope of the calibration curve, and the limit of detection (LOD) was calculated at Signal to Noise ratio (S/N) = 3. The sensitivity and LOD were evaluated in the linear range of the calibration curve.

## 3. Results and Discussion

### 3.1. Ag and Au NPs’ Preparation and Characterization

The procedure for the preparation of Ag and Au NPs is illustrated in Scheme 1. First, the methanolic extract of *Rumex roseus* was obtained following the procedure reported in our previous work [16].

**Figure 1:**
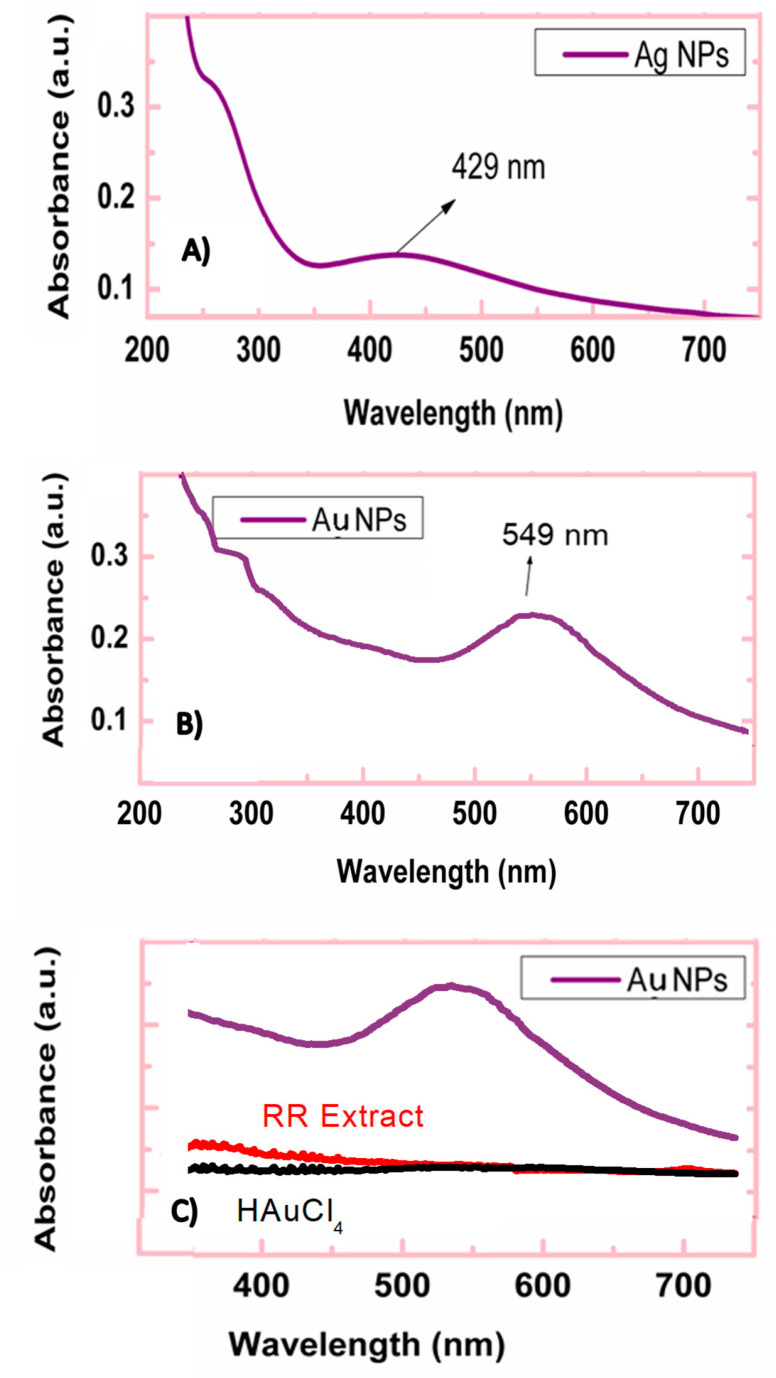
UV-Vis spectra recorded in the reaction solution for the synthesis of (**A**) AgNPs and (**B**) AuNPs. Plasmonic peaks assigned to metal nanoparticles are indicated by arrows. (**C**) UV-Vis spectra of the *Rumex roseus* (RR) plant extract and Au metal precursor are also shown.

The formation of silver and gold nanoparticles was monitored by UV-Vis measurement of the reaction solution (see Figure 1A–C). The strong absorption in the UV range between 200 and 400 nm is due to the presence of methanol and organic compounds in the extract of *Rumex roseus*. A similar finding was found for other plant extracts during the synthesis of AgNPs and AuNPs [15,31]. At higher wavelengths, the adsorption is due to the plasmonic peak of Ag and Au nanoparticles because no adsorption in this range was noted for the RR plant extract and metal precursors.

As regards AgNPs synthesis, in the reaction solution after 2 h of reaction, a plasmonic peak with a maximum of absorbance at a wavelength of 429 nm was found, which confirms the formation of Ag nanoparticles at nanoscale. The broad plasmonic band suggests that they are poly-dispersed. From the relationships between Ag plasmon peak wavelength and particle size [32], the average value for the Ag size was calculated to be 58 nm.

A plasmonic band at 549 nm has also been recorded in the case of AuNPs. The presence of a single plasmonic peak suggests that Ag and Au nanoparticles have an almost spherical shape according to Mie theory [33]. Other authors reported similar findings by using different plant extracts [34,35]. A confirmation of this has also been reported for AuNPs synthesized by us using *Rhanterium suaveolens* plant extract in similar operative conditions [36]. The particle size for the AuNPs sample, derived from the Au plasmonic band, was found to be 80 nm [37]. No remarkable variation has been observed registering the UV-Vis spectrum after 24 h from the NPs synthesis, confirming the stability of the colloidal solution containing the Ag and Au NPs.

**Figure 2:**
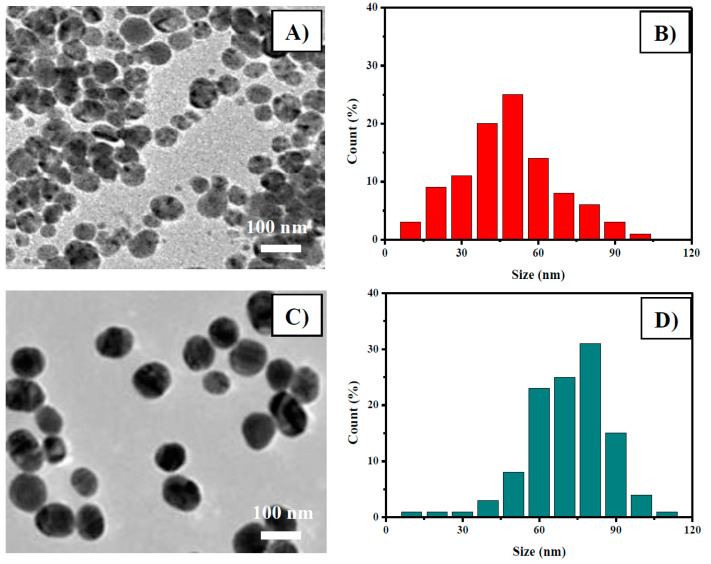
Transmission electron microscopy (TEM) of (**A**) AgNPs and (**C**) AuNPs. Particle size distribution of (**B**) AgNPs and (**D**) AuNPs.

TEM analysis was performed to determine the size and shape of the obtained Ag and Au nanoparticles. The round shape of Ag nanoparticles, foreseen by UV-Vis analysis, was also confirmed by TEM analysis (see Figure 2A). The average diameter of AgNPs was calculated to be 50 ± 45 nm. For the AuNPs sample, larger particles with a round shape and an average diameter of 75 ± 30 nm have been found (Figure 2B). The particle size distribution for both samples, derived by counting an elevated number of particles (>150), is reported in Figure 2C,D.

**Figure 3:**
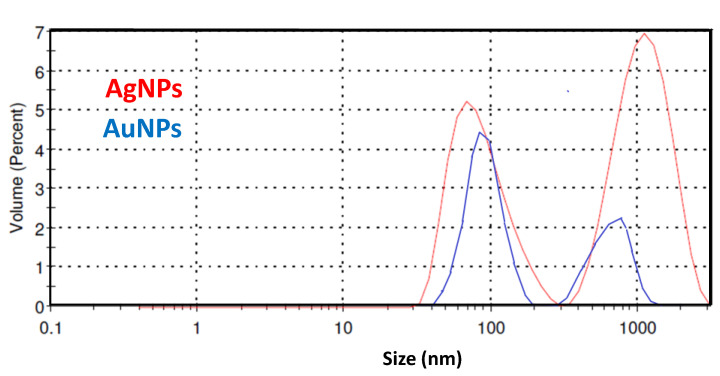
Dynamic light scattering (DLS) pattern for AgNPs and AuNPs.

DLS was also used for analyzing the size distribution of the synthesized Ag and Au nanoparticles in aqueous suspension. Differently from UV-Vis and TEM data, by this technique, we can determine the secondary particle size of nanoparticles dispersed in a specific polar or non-polar solvent [38]. DLS spectra of AgNPs and AuNPs redispersed in the aqueous solution are shown in Figure 3. The plot shows the volume (percent) of particle light scattering against particle size. The two distinct peaks observed at 74 and 1100 nm clearly indicate a bimodal distribution, signaling the polydispersities of the AgNPs in the aqueous dispersion.

The formation of these aggregates is likely due to the interaction between the AgNPs and plant extract biomolecules which completely surround the metal core, consequently leading to an increase of the hydrodynamic diameter.

The particle size distribution of non-aggregated AgNPs is centered around 74 nm, which is larger than those measured by the other techniques used here. This finding is usually reported when using DLS for measuring particle sizes. Indeed, as the larger particles scatter the majority of the light, aggregate species have greater influence than primary particles [38].

As in the case of AgNPs, the pattern of AuNPs shows a bimodal distribution. The first peak is centered at around 85 nm, whereas the second peak is shifted at lower particle size compared to the corresponding peak found on AgNPs and furthermore displays a weaker intensity, indicating a lower particle agglomeration. The comparison of the AgNPs and AuNPs particle size distribution, derived by TEM and DLS analyses, confirms the above findings.

### 3.2. Electrochemical Characteristics of Ag-NPs/GCE

**Figure 4:**
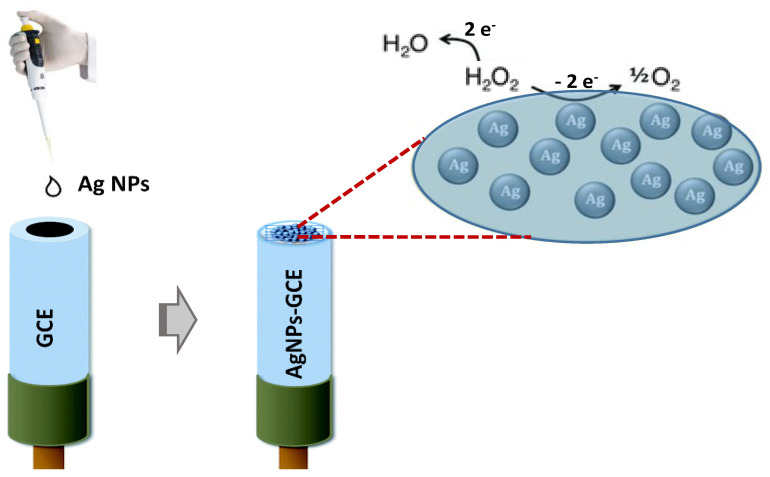
Schematization of the fabrication of modified AgNPs/glassy carbon electrode (GCE). The electrochemical reactions which can occur in the presence of H_2_O_2_ are highlighted.

Figure 4 displays a schematization of the modified electrochemical sensor fabrication, performed by drop-coating of the modifiers on the top surface of the GCE, and its general functioning principle for the determination of H_2_O_2_, relying on the oxidation or reduction of this analyte. The electrochemical performances of AgNPs- and AgNPs-rGO-modified GCEs towards reduction of H_2_O_2_ were evaluated here.

**Figure 5:**
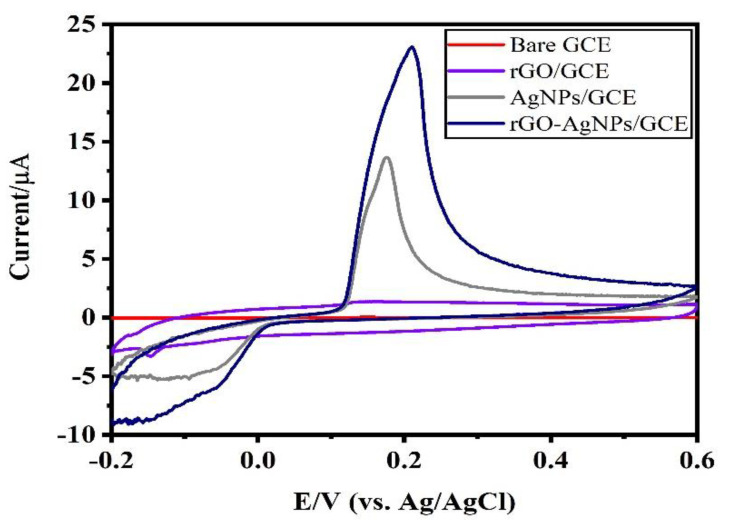
Electrochemical behavior of bare GCE, reduced graphene oxide (rGO)/GCE, AgNPs/GCE and AgNPs-rGO/GCE in air-saturated 0.1 M HCl electrolyte at a scan rate of 50 mV/s.

The behavior of the bare GCE, rGO/GCE, AgNPs/GCE and AgNPs-rGO/GCE was evaluated by cyclic voltammetry (CV) in air-saturated 0.1 M HCl solution as electrolyte at a scan rate of 50 mV/s (Figure 5). Bare GCE and rGO/GCE did not show any anodic or cathodic current peak in their respective voltammograms due to the absence of redox reaction on the surface of the electrode.

Instead, GCEs modified with AgNPs and AgNPs-rGO composite exhibited well-pronounced anodic peaks at around 0.2 V, associated with the oxidation of the Ag^0^ into Ag^+^, while the reduction (cathodic) peak refers to the reduction of Ag^+^ to Ag^0^ [39]. The AgNPs-rGO composite-modified GCE showed the highest redox couple intensity, due to high dispersion of the AgNPs on the rGO support and the enhanced conductivity [40]. It was also noted that there is a peak shift towards positive potential for the AgNPs-rGO composite when compared to that of AgNPs. The observed anodic peak shift and broad nature of the peaks of AgNPs and AgNPs-rGO composite might be due to the formation of Ag_2_O at a potential close to the that of AgO.

**Figure 6:**
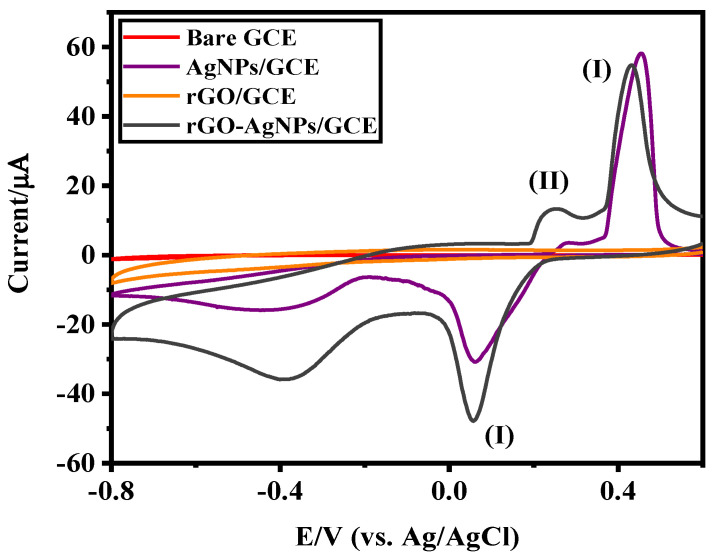
Cyclic voltammograms (CV) of bare GCE and rGO-, AgNPs- and rGO-AgNPs-modified GCEs in the presence of 300 µM H_2_O_2_ in 0.1 M phosphate buffer solution (PBS) (pH 7.0) and (I) denotes the reaction of formation of AgO and (II) denotes the formation of Ag_2_O during the potential scan.

The cyclic voltammetric responses of the bare GCE, AgNPs/GCE, rGO/GCE and AgNPs-rGO/GCE in the presence of 300 µM H_2_O_2_ in 0.1 M PBS (pH 7.0) are shown in Figure 6.

It can be noticed that the rGO-AgNPs-modified GCE exhibits a strong reduction peak of H_2_O_2_ [19] at a potential of about −0.40 V when compared to other electrodes such as bare GCE, Ag NPs/GCE and rGO/GCE. In addition, a pair of redox peaks was evidenced at 0.45 and 0.054 V respectively, due to the formation and reduction of AgO (reaction I). A small satellite oxidation peak appears at about 0.25 V corresponding to the formation of Ag_2_O (reaction II) [41]. This indicates the presence of mixed compounds AgO and Ag_2_O, the major composition being AgO.

(I) $$Ag_{2}O+2OH^{-}\to 2AgO+H_{2}O+2e^{-}$$

(II) $$2Ag+2OH^{-}\to Ag_{2}O+H_{2}O+2e^{-}$$

**Figure 7:**
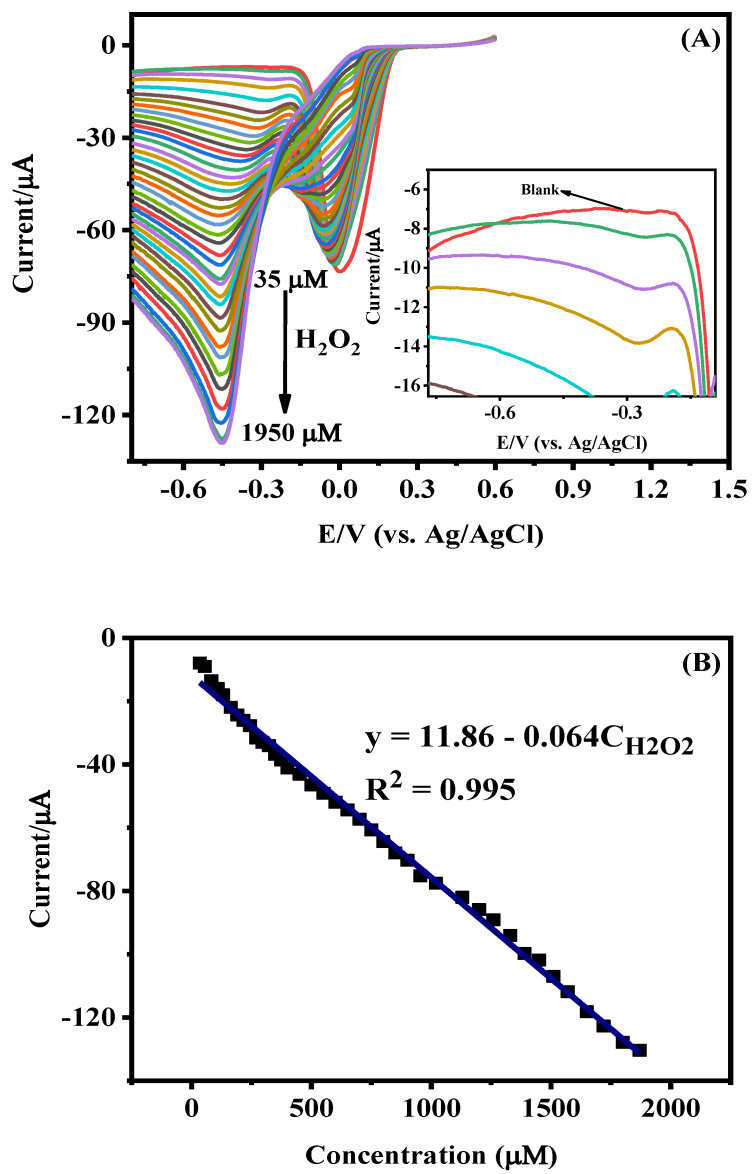
(**A**) CV of AgNPs-rGO/GCE recorded at a scan rate of 50 mV/s at different concentrations of H_2_O_2_ (from 35 µM to 1.95 mM) in 0.1 M PBS (pH 7.0). (**B**) Calibration curve for H_2_O_2_, determined taking into account the cathodic peak current at −0.45 V. The standard deviation of response calculated from data in the calibration curve is 2.464 μA.

Cyclic voltammetric studies with rGO-AgNPs-modified GCE were carried out for various concentrations (35 to 1950 μM) of 1 mM H_2_O_2_ at pH 7.0. CVs recorded after applying baseline correction are shown in Figure 7A. The peak observed at 0.03 V, corresponding to the reduction of Ag, was found to decrease its intensity with the increase in the concentration of H_2_O_2_. Initially, the reduction peak of H_2_O_2_ appeared at −0.28 V for lower concentrations of 35 µM to 400 µM, and the peak intensity increased with the increase in the H_2_O_2_ content and reached the maximum at 1950 μM. Moreover, a small but systematic negative shift in the peak potential was also observed as a function of the H_2_O_2_ content. This behavior can be explained as follows. Actually, the observed cathodic peak current is a direct measure of the rate of the electrochemical reaction taking place at the electrode surface. At low concentrations of the analyte, mass diffusion occurs steadily, but at high concentrations, the diffusion process is disrupted due to the deposition of reaction products of H_2_O_2_ on the electrode surface. In this condition, the electrochemical system requires higher potential, resulting, therefore, in the shift in the peak potential from −0.28 to −0.45 V with the increase in H_2_O_2_ concentration.

The calibration curve (Figure 7B) was plotted by taking all the peak currents as a function of concentration, which resulted in the regression equation y = 11.86 − 0.064 × C_H2O2_. The slope over the linear range of 35 µM to 1.95 mM was used to calculate the sensitivity as 64 mA mM^−1^, and the lowest detection limit of 1.1 µM with the signal-to-noise (S/N) ratio of 3.

**Figure 8:**
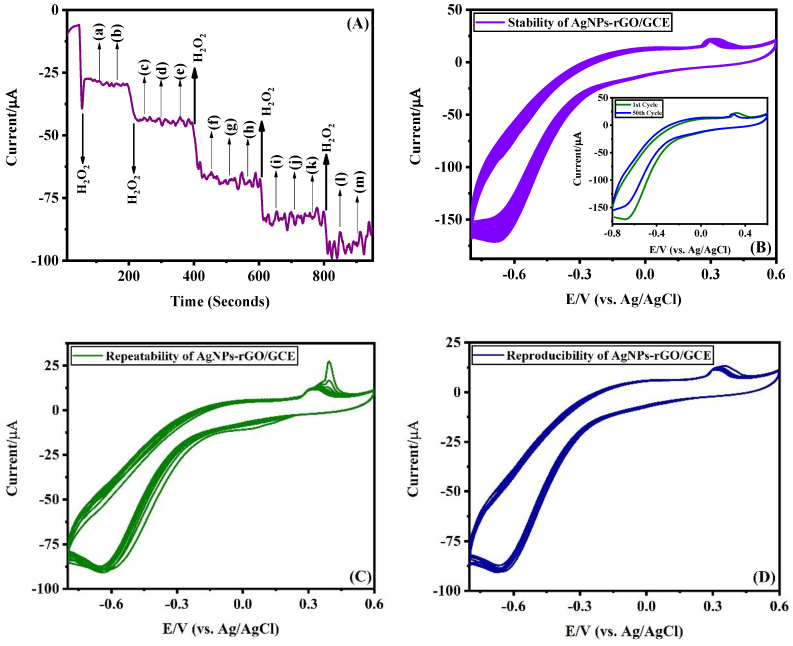
(**A**) Anti-interference ability of rGO-AgNPs/GCE in the presence of 50-fold excess of (a) Na^+^, (b) K^+^, (c) NH_4_^+^, (d) Mg^2+^, (e) Cl^−^, (f) SO_4_^2−^, (g) glucose, (h) urea, (i) nitrite, (j) serotonin, (k) tyrosine, (l) dopamine and (m) uric acid is recorded by the chronoamperometric method in 0.1 M PBS (pH 7.0). (**B**) Cyclic voltammograms recorded for 50 cycles at rGO-AgNPs/GCE in 800 µM of H_2_O_2_ in 0.1 M PBS (pH 7.0) at a scan rate of 50 mV/s and inset shows the CVs of first cycle and 50th recorded at AgNPs modified GCE. (**C**) Repeatability study was performed by making ten repetitive CV measurements at rGO-AgNPs/GCE in 700 µM of H_2_O_2_ in 0.1 M PBS (pH 7.0) at a scan rate of 50 mV/s. (**D**) CVs performed for reproducibility of 10 different GCEs modified with rGO-AgNPs in 700 µM H_2_O_2_ in 0.1 M PBS (pH 7.0) at a scan rate of 50 mV/s.

The anti-interference ability of the electrode has been tested by the chronoamperometry method in the presence of 50-fold excess of potential interferents such as Na^+^, K^+^, NH_4_^+^, Mg^2+^, Cl^−^, SO_4_^2−^, glucose, urea, nitrite, serotonin, tyrosine, dopamine and uric acid (Figure 8A). From the results, it is observed that these interferents have no effect on H_2_O_2_ detection.

The cyclic stability of the rGO-AgNPs/GCE was studied by recording 50 cycles of CVs in 0.1 M PBS (pH 7.0) containing 800 μM of H_2_O_2_ at a scan rate of 50 mV/s and the results are shown in Figure 8B. As shown in the insert of Figure 8B, there is a small decrease in reduction peak current of H_2_O_2_ which confirms the good cyclic stability of the electrode. The current values from the first cycle and the 50th cycle were noted and used to calculate the Relative Standard Deviation (RSD) value, which is found to be 8.2%.

The repeatability of the fabricated rGO-AgNPs/GCE was investigated by conducting a series of ten repetitive measurements using the same electrode in 0.1 M PBS (pH 7.0) containing 700 μM H_2_O_2_. The peak current due to the reduction of H_2_O_2_ at each measurement was noted and the RSD value was calculated as 2.8%. In order to study the reproducibility of the fabricated sensor, ten different rGO-AgNPs-modified GCEs were prepared under identical conditions. CVs were recorded for all the modified electrodes in 0.1 M PBS (pH 7.0) solution containing 700 μM H_2_O_2_. The value of RSD was calculated by noting the reduction current at each CV and it was found to be 2.81%.

### 3.3. Electrochemical Characteristics of AuNPs/SPCE

The suspension containing the AuNPs was also used to fabricate AuNPs-modified electrode screen-printed carbon electrodes. Using SPCE presents many advantages compared to GCE, such as low cost, simpler operation and easy mass production using thick film technology [42]. These advantages have been extensively used for developing novel electrochemical sensing platforms for practical applications [43]. Indeed, screen-printed electrodes can be used as disposable devices. Further, as the working electrode is printed into a very small area, the electrode materials needed for its fabrication are minimal. Consequently, screen-printed sensors are cheaper and can be manufactured in high volume at very low cost.

**Figure 9:**
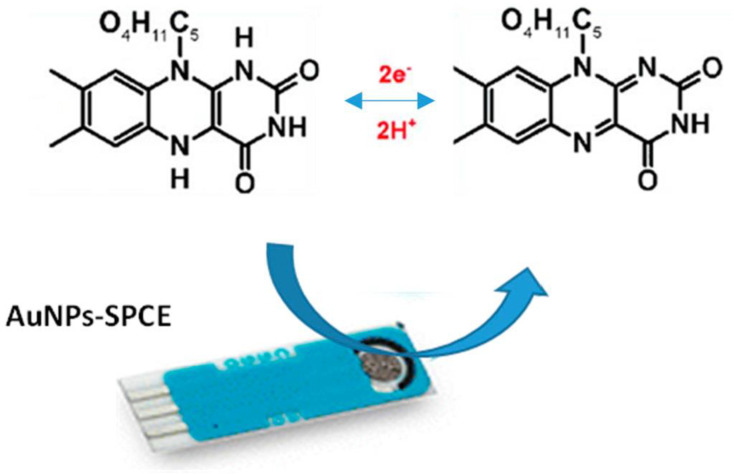
Picture of the modified AuNPs-screen-printed carbon electrodes (SPCE) and representation of the electrochemical reactions exploited for the detection of riboflavin (RF).

Here, we report data on the investigation of the electrochemical characteristics of the AuNPs/SPCE for the electroanalytical determination of RF. A picture of the modified AuNPs-SPCE and the representation of the reversible electrochemical oxidation-reduction of riboflavin, involving a two-electron process, is reported in Figure 9.

Due to the reversible oxidation and reduction reactions involving RF, both of these processes can be investigated over the most commons electrodes, depending on the potential conditions applied. Most of the studies on the electrochemical detection of RF relies on the cathodic reduction on the electrode surface [44]. However, many investigations also report the electrochemical detection of RF through the anodic oxidation [45,46,47].

**Figure 10:**
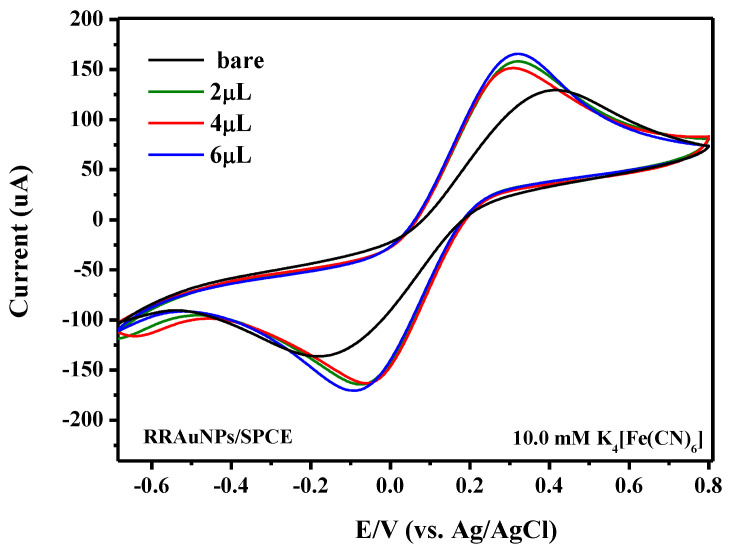
Cyclic voltammograms of bare SPCE and modified SPCE with different loading of dipped AuNPs. Scan rate 50.0 mV/s in 0.1 M PBS solution containing 10.0 mM K_4_[Fe(CN)_6_].

First, we evaluated the effect of AuNPs on the electrochemical characteristics of the bare SPCE platform by cyclic voltammetry (Figure 10), by using ferrocyanide (10 mM K_4_[Fe(CN)_6_]) as a probe, in 1 M phosphate buffer solution (PBS) at a scan rate of 50 mV/s. CVs of modified AuNPs/SPCE show the characteristic redox couple of ferrocyanide which appears to be increased in intensity compared to bare SPCE; furthermore, the peak difference is somewhat reduced (354 mV), suggesting that AuNPs help the electron transfer reaction and also favor the redox reaction. As regarding the AuNPs’ loading, a volume of 2 µL of AuNPs (having a concentration of 5 mg/mL) dipped in SPCE has been demonstrated to be sufficient in order to obtain the maximum response. By considering the result obtained, the SPCE modified with 2 µL of AuNPs solution was used in the subsequent experiments.

The determination of electrochemical active area (ECSA) values is of great importance to obtain a comparison of the performances between different electrodes and can provide helpful indication about changes at the electrode interface after modification. Indeed, the observed improvement in redox peak current values of AuNPs-based electrodes could be attributed to faster electron transfer kinetics and its larger electroactive surface area. The Randles–Sevcik formula was employed to find out the active surface area of the SPCE and AuNPs/SPCE by using the K_4_[Fe(CN)_6_]/K_3_[Fe(CN)_6_] as a redox probe:

*I*_p_*=* 2.69 × 10^5^*n*^3/2^*AD*^1/2^*C υ*^1/2^ (1)

where *I*_p_ is the current peak measured at redox potential, *n* = 1 is the number of electrons transferred in the redox event, A = 0.126 cm^2^ is the electrode surface area (cm^2^), D = 7.6 × 10^−6^ cm^2^ s^−1^ is the diffusion coefficient (cm^2^ s^−1^), C is the redox probe concentration (in the range 1 to 10 mM (10^−6^ to 10^−5^ mol cm^3^)) and *v* = 0.05 is the potential scan rate (Vs^−1^). From the slope of the Equation (1), the calculated areas for SPCE and AuNPs/SPCE were found to be 0.238 and 0.346 cm^2^ respectively, confirming that the modified AuNPs/SPCE provides a 50% larger ECSA than bare SPCE.

**Figure 11:**
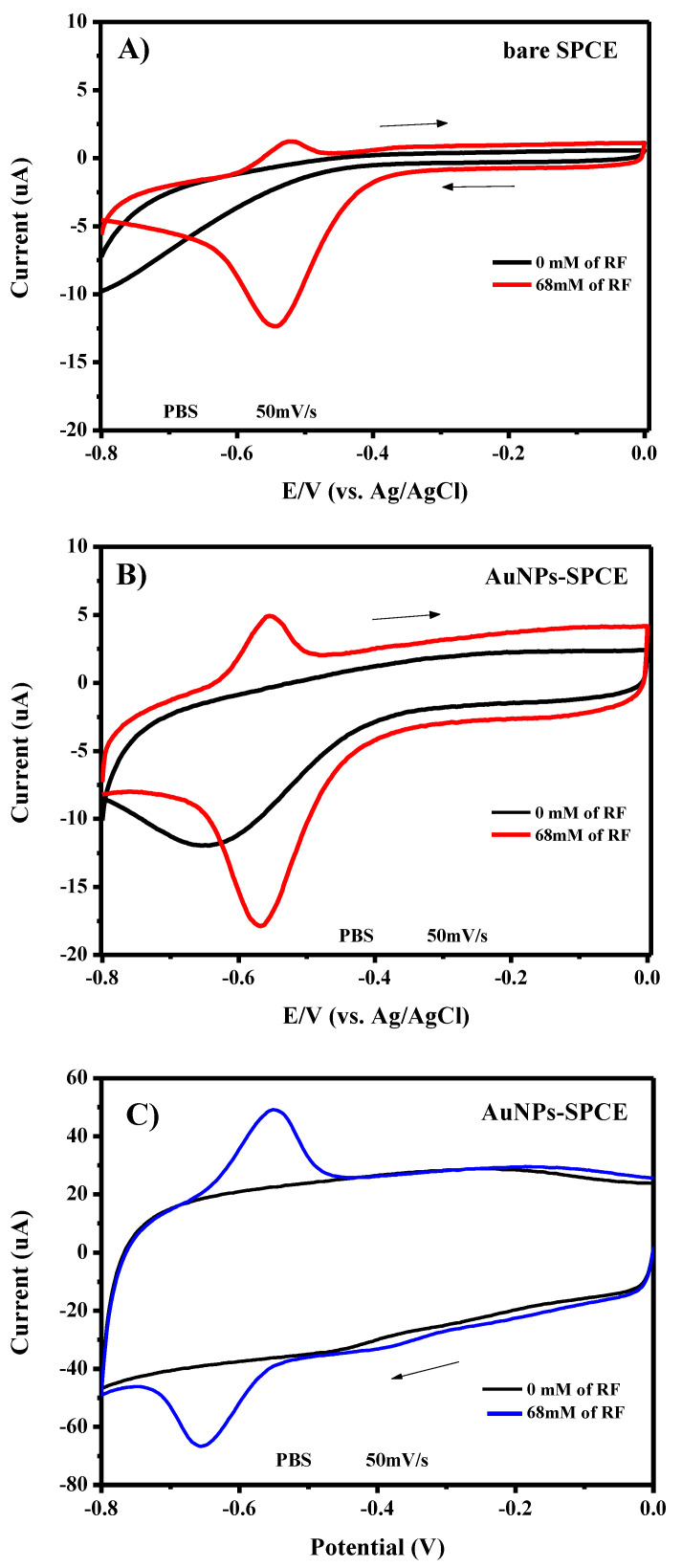
Electrochemical behavior of (**A**) bare SPCE and (**B**,**C**) AuNPs-modified electrode in the absence and presence of riboflavin. CV was performed in de-aerated 1 M PBS at a scan rate of 50 mV/s. The arrows indicate the direction of the starting potential scan.

In order to find the optimal conditions for RF determination with the developed AuNPs/SPCE, we carried out a series of tests in the absence and presence of riboflavin, and changing the potential direction in both the positive or negative direction. Figure 11A,B compare CVs obtained for the bare SPCE and modified AuNPs/SPCE respectively, in the absence and presence of riboflavin in 1 M PBS, changing the potential in the positive direction, starting from the potential value of −0.8 to 0.0 V. Many differences between SPCE and modified AuNPs/SPCE can also be observed in the absence of riboflavin. The most important is related to the greater capacitive current registered on the modified AuNPs/SPCE, as clearly evidenced by the larger CV cycle compared to the narrow cycle of the bare electrode. Such behavior can be explained with the increased surface area of the working electrode introduced after AuNPs’ modification. Further, in the modified electrode, a large cathodic peak is present at around −0.65 V. We supposed that this peak can be due to cathodic reduction of residual O_2_ in the electrolyte solution. The CV curve of AuNPs/SPCE in the presence of RF is characterized by the presence of a pair of peaks at −0.551 and −0.567 V, with a peak-to-peak separation (Δ*E*_p_) of only 16 mV, which is indicative of a reversible oxidation/reduction process of RF on the electrode surface. However, the anodic peak displays a very low intensity compared to the cathodic peak.

Figure 11C reports the tests carried out as above but changing the potential in the negative direction, starting from the potential value of 0 to −0.8 V. A redox pair of high intensity was obtained at −0.548 and −0.651 V, with a Δ*E*_p_ of 103 mV. The ratio of redox peak currents (*I*_pa_/*I*_pc_) was ≈0.9, which is a characteristic of the quasi-reversible electrode process. From these tests, it appears that the latter procedure is more effective for obtaining better performances, so it was considered for the electroanalytical tests for the determination of riboflavin with the modified AuNPs-SPCE.

**Figure 12:**
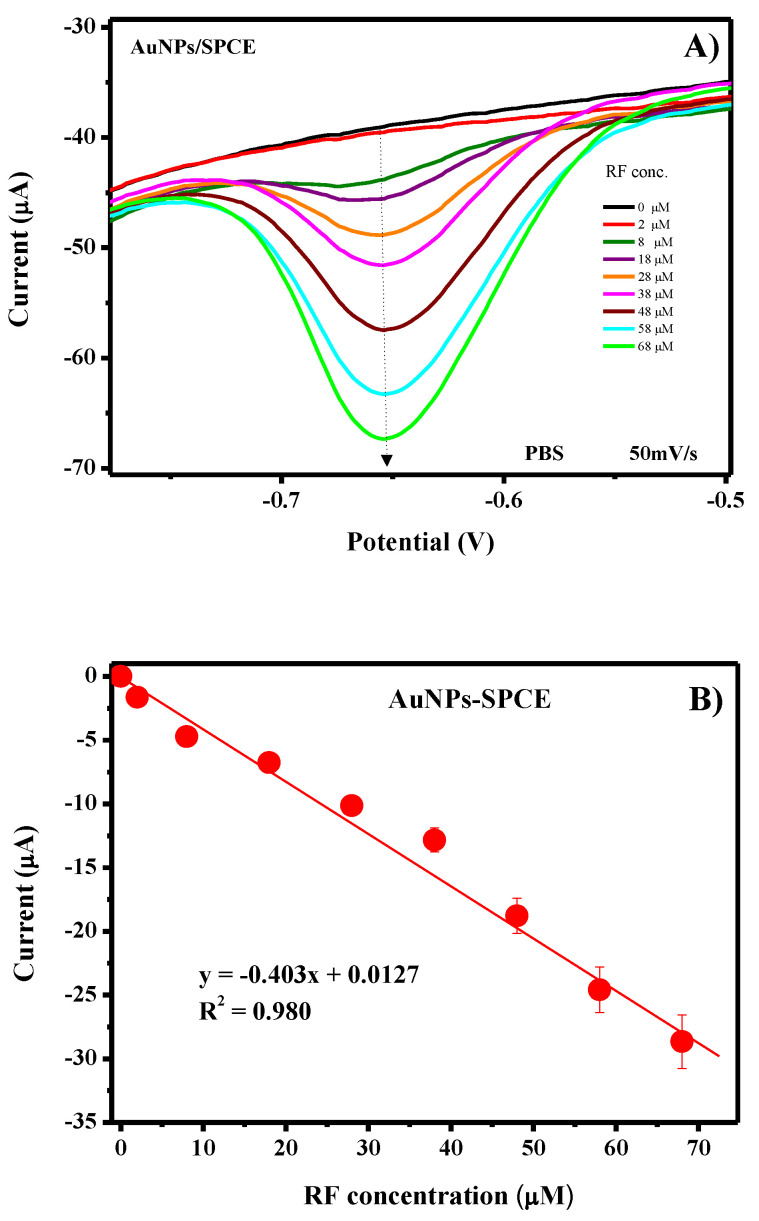
(**A**) Linear Sweep Voltammetry (LSV) of AuNPs/SPCE, performed in 0.1 M PBS electrolyte and in the presence of different concentrations of RF. (**B**) Calibration curve for the determination of RF. The standard deviation of response calculated from data in the calibration curve is 7.623.

In Figure 12A, the Linear Sweep Voltammetry (LSV) analysis with the modified AuNPs-SPCE of different concentrations of riboflavin (from 0 to 70 µM) is reported. Experimental conditions are the same as applied to obtain the CV curves. Well-defined LSV curves have been reported, allowing to derive the calibration curve reported in Figure 12B. First, the stability of the LSV measurement obtained with the AuNPs/SPCE sensor has been confirmed by the low RSD value (<3.5%) computed on a series of 25 repeated measurements in the presence of 500 μM of riboflavin. Further, no remarkable variation of the anodic peak current and potential was observed after a long period of use (3–4 months), indicating that no or little deterioration of the electrode performances occurred also in an extended period. The reliability of the sensor was evaluated by the determination of RF concentration by repetitive measurements (*n* = 3) with the same electrode. The RSD for 50 μM RF determination was found to be less than 5%, demonstrating its good signal reproducibility. A well-linear trend was observed in the range 0–70 µM. The linear regression equation was deduced as y = −0.403*x* + 0.0127 with a R^2^ value of 0.980 and LOD was calculated as 1.3 μM at S/N = 3.

A verification of the literature data (compare Table 1 in Reference [48]) indicates that our developed sensor shows characteristics close to the state-of-the-art riboflavin sensors. These performances demonstrate that the modification of the SPCE with gold nanoparticles has introduced a lot of advantages in terms of higher sensitivity and lower detection limits towards riboflavin with respect to bare electrodes.

## 4. Conclusions

In summary, the electrochemical behavior of Ag and Au NPs obtained by using *Rumex roseus* plant and finalized to the development of new modified electrochemical sensors was reported here. It was demonstrated that the phytochemicals (in particular, the polyphenols fraction) contained in the *Rumex roseus* extract act effectively as reducing agents for the Ag^+^ and Au^+3^ ions. The reduction process led to the formation of nearly spherical Ag and Au nanoparticles. Glassy carbon electrode and screen-printed carbon electrode have been modified by these nanoparticles and the electrochemical and electroanalytical performances for the hydrogen peroxide and riboflavin determination have been investigated. The modified electrodes greatly improved the current response to these target analytes and resulted in good sensing performance. The promising preliminary results of this study encourage us to continue in the search and optimization of the method of preparation of metal nanoparticles by plant extracts for developing novel electrochemical sensors.
